# High-sensitive cardiac troponin T and NT-proBNP are associated with the left ventricular apical thickness in apical hypertrophic cardiomyopathy

**DOI:** 10.1186/s40001-024-02222-7

**Published:** 2025-01-23

**Authors:** Meng Zhang, Wei Gao, Xiaotong Cui, Xueting Han, Yamei Xu, Jingmin Zhou, Junbo Ge

**Affiliations:** 1 Department of Cardiology, Zhongshan Hospital, Fudan University (Xiamen Branch), Xiamen, China; 2 Department of Cardiology, Zhongshan Hospital, Fudan University, Shanghai Institute of Cardiovascular Diseases, Shanghai, China; 3 National Clinical Research Center for Interventional Medicine, Shanghai, China

**Keywords:** Apical hypertrophic cardiomyopathy, High-sensitive cardiac troponin T, N-terminal pro-BNP, Left ventricular apical thickness

## Abstract

**Background:**

Apical hypertrophic cardiomyopathy (AHCM) is a subtype of hypertrophic cardiomyopathy (HCM). The expression level of high-sensitive cardiac troponin T (hs-cTNT) and N-terminal pro-BNP (NT-proBNP) in AHCM patients, and these relationships between echocardiography parameters were still unclear.

**Methods:**

We retrospectively screened AHCM patients between January 2019 and December 2021 in Zhongshan Hospital Fudan University. The relationship between the level of hs-cTNT, NT-proBNP and echocardiography parameters were analyzed. The risk factors for elevated hs-cTNT and NT-proBNP level were investigated with linear regression analysis.

**Results:**

A total of 267 AHCM patients were enrolled. They were divided into hs-cTNT normal (129, 48.3%) and abnormal (138, 51.7%) group. Compared with hs-cTNT normal group, hs-cTNT abnormal group were elder (68.3 ± 11.6 vs. 63.8 ± 10.6, *P* = 0.001); with higher rate of atrial fibrillation (AF) (41.3% vs. 17.8%, *P* < 0.001) and higher level of NT-proBNP concentration (752.0 [343.8–1345.5] vs. 249.0 [104.0–541.0], *P* < 0.001). For echocardiography parameters, hs-cTNT abnormal patients have thicker interventricular septum (IVS) (11.6 ± 2.0 vs. 11.0 ± 1.7, *P* = 0.02), thicker left ventricular apical (LVA) (16.9 ± 3.0 vs. 14.9 ± 2.3, *P* < 0.001) and larger left atrium diameter (LAD) (45.9 ± 6.6 vs. 42.4 ± 5.1,* P* < 0.001). LVA was independently correlated with both the level of hs-cTNT and NT-proBNP (hs-cTNT *r* = 0.224, *P* = 0.143; NT-proBNP *r* = 0.370, *P* < 0.001). Linear regression analysis revealed that LVA was independent risk factor of both the elevated hs-cTNT and NT-proBNP level.

**Conclusions:**

More than half of AHCM patients had abnormal hs-cTNT level. LVA was positively and independently correlated with the level of hs-cTNT and NT-proBNP.

## Introduction

Apical hypertrophic cardiomyopathy (AHCM), a subtype of hypertrophic cardiomyopathy (HCM), charactered with apical hypertrophic and precordial giant negative T-wave (GNT), was described in 1976s for the first time [1]. Previous reports showed that AHCM, when with symptom of chest pain or dyspnea, elevated cardiac markers and GNT, was highly suspected as non-ST-elevation myocardial infarction (NSTEMI) [2–4]. This has strong implications for diagnosis and treatment. It is thus important to investigate the differentiated level and clinical value of high-sensitivity cardiac troponin T (hs-cTnT) and N-terminal brain natriuretic peptide (NT-proBNP) in the AHCM. Elevated serum hs-cTnT and NT-proBNP were commonly found in patients with HCM, associated with left ventricular (LV) wall thickness, extension of myocardial fibrosis and adverse cardiovascular events [5–7]. However, the expression level and clinical value of hs-cTnT and NT-proBNP in patients with AHCM were rarely reported.

The aim of our study was to describe the expression of hs-cTNT and NT-proBNP in AHCM patients, and identify their associations with the echocardiography parameters, especially the left ventricular apical thickness.

## Methods

### Study design and patient selection

We retrospectively screened pure AHCM patients between January 2019 and December 2021 in Zhongshan Hospital Fudan University. The diagnostic criterion for AHCM was based on echocardiography, contained LV hypertrophy was restricted to the LV apex and maximal apical wall thickness of ≥ 15 mm or a ratio of maximal apical to posterior wall thickness of ≥ 1.5 [8, 9]. They were divided into two groups (hs-cTNT normal group and hs-cTNT abnormal group) on the basis of hs-cTNT level. The criterion for hs-cTNT abnormal is above 0.014 ng/mL. Patients with acute myocardial infarction; heart failure with New York Heart Association (NYHA) class III or IV; modest to severe valve disease; abnormal ventricular wall motion and eGFR levels below 30 mL/(min/1.73 m^2^) were excluded from this study.

**Fig. 1:**
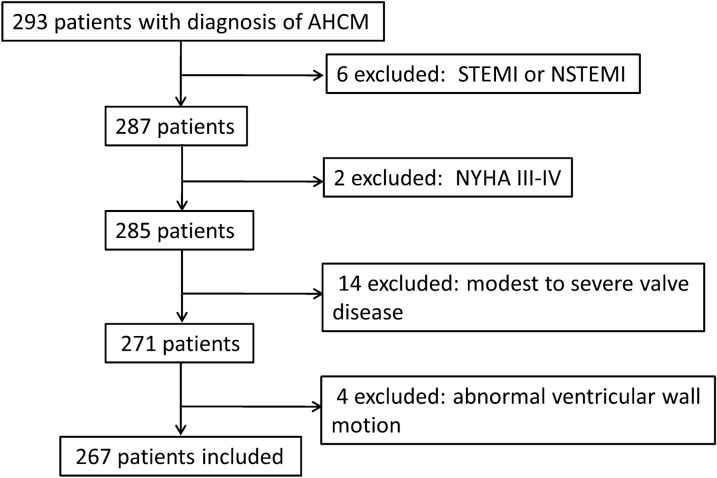
Flow chart of included patients

As shown in the flowchart in Fig. 1, a total of 293 patients were screened and 26 patients were excluded for the following reasons: acute myocardial infarction (*n* = 6); heart failure with NYHA class III or IV (*n* = 2); modest to severe valve disease (*n* = 14) and abnormal ventricular wall motion (*n* = 4). Finally, 267 patients were included in this study, which was approved by the hospital's Ethics Committee.

### Laboratory testing and echocardiography

A venous blood sample was collected for all patients at admission. All the laboratory assays were performed by the central laboratory at our hospital. Hs-cTnT and NT-proBNP concentration were detected at admission. Echocardiography was performed within 24 h after admission and left ventricular ejection fraction (LVEF), left atrium diameter (LAD), interventricular septum thickness (IVS), left ventricular apical thickness (LVA), left ventricular end-diastolic diameter (LVEDD) were determined.

### Statistical analysis

Normally distributed data are expressed as mean ± SD, and were compared using the independent-samples *T* test. Skewed variables are expressed as median and inter quartile range and Mann–Whitney *U* test was used. Categorical data are expressed as number (percentage) and were compared using the chi-squared test. The correlation test was undertaken with the linear correlation analysis. Multivariable linear regression was used to evaluate risk factors. All statistical analyses were performed using SPSS 22.0 (SSPS Inc., Chicago, IL, USA). A value of *P* < 0.05 was considered as statistically significant.

## Results

### Characteristics of patients studied

A total of 267 patients were included in the present study and their characteristics are summarized in Table 1. They were divided into hs-cTNT normal group (*N* = 129, 48.3%) and hs-cTNT abnormal group (*N* = 138, 51.7%). Compared with hs-cTNT normal group, hs-cTNT abnormal group were elder (68.3 ± 11.6 vs. 63.8 ± 10.6, *P* = 0.001); more likely to be suffered with atrial fibrillation (AF) (41.3% vs. 17.8%, *P* < 0.001) and with higher level of NT-proBNP concentration (752.0 [343.8–1345.5] vs. 249.0 [104.0–541.0], *P* < 0.001). For echocardiography parameters, hs-cTNT abnormal patients had thicker IVS (11.6 ± 2.0 vs. 11.0 ± 1.7, *P* = 0.02) and LVA (16.9 ± 3.0 vs. 14.9 ± 2.3, *P* < 0.001), larger LAD (45.9 ± 6.6 vs. 42.4 ± 5.1,* P* < 0.001). Furthermore, it should be underscored that the presence of current coronary artery disease (CAD) did not have statistical significance in two groups (20.9% vs. 13.8%, *P* = 0.121).

**Table 1:** Characteristics of the study population by hs-cTnT level

	Hs-cTNT normal (*N* = 129)	Hs-cTNT abnormal (*N* = 138)	*P* value
Age (years)	63.8 ± 10.6	68.3 ± 11.6	0.001
Male (*n*, %)	91 (70.5%)	103 (74.6%)	0.453
Smoking (*n*, %)	37 (28.7%)	37 (26.8%)	0.733
Hypertension (*n*, %)	77 (59.7%)	83 (60.1%)	0.940
Diabetes (*n*, %)	28 (21.7%)	33 (23.9%)	0.668
AF (*n*, %)	23 (17.8%)	57 (41.3%)	< 0.001
Presence of current CAD (*n*, %)	27 (20.9%)	19 (13.8%)	0.121
Hs-cTNT (ng/mL)	10.0 [8.0–12.0]	24.0 [19.0–34.0]	< 0.001
NT-proBNP (pg/mL)	249.0 [104.0–541.0]	752.0 [343.8–1345.5]	< 0.001
CK (U/L)	90.0 [65.0–120.0]	93.5 [66.0–140.8]	0.220
CK–MB (U/L)	15.0 [12.0–17.0]	16.0 [12.7–19.2]	0.207
AST (U/L)	22.0 ± 7.8	23.4 ± 11.4	0.289
ALT (U/L)	26.2 ± 14.6	22.9 ± 12.4	0.061
Creatinine (mmol/L)	79.4 ± 16.1	93.8 ± 30.4	< 0.001
eGFR [mL/(min 1.73 m^2^)]	84.6 ± 15.1	72.9 ± 21.1	0.008
Hb (g/L)	142.2 ± 13.8	137.0 ± 17.2	< 0.001
IVS (mm)	11.0 ± 1.7	11.6 ± 2.0	0.020
LVA (mm)	14.9 ± 2.3	16.9 ± 3.0	< 0.001
LAD (mm)	42.4 ± 5.1	45.9 ± 6.6	< 0.001
LVEDD (mm)	48.4 ± 3.9	48.8 ± 4.2	0.441
LVEF (%)	65.5 ± 3.9	65.0 ± 4.7	0.285

hs-cTNT: high-sensitive cardiac troponin T; NT-proBNP: N-terminal pro-BNP; CK: creatine kinase; CKMB: creatine kinase-MB; AST: aspartate aminotransferase; ALT: alanine aminotransferase; eGFR: estimated glomerular filtration rate; Hb: hemoglobin; AF: atrial fibrillation; CAD: coronary artery disease; IVS: interventricular septum thickness; LVA: left ventricular apical thickness; LAD: left atrium diameter; LVEDD: left ventricular end-diastolic diameter; LVEF: left ventricular ejection fraction

### Relationship between hs-cTnT, NT-proBNP levels and LVA

**Fig. 2:**
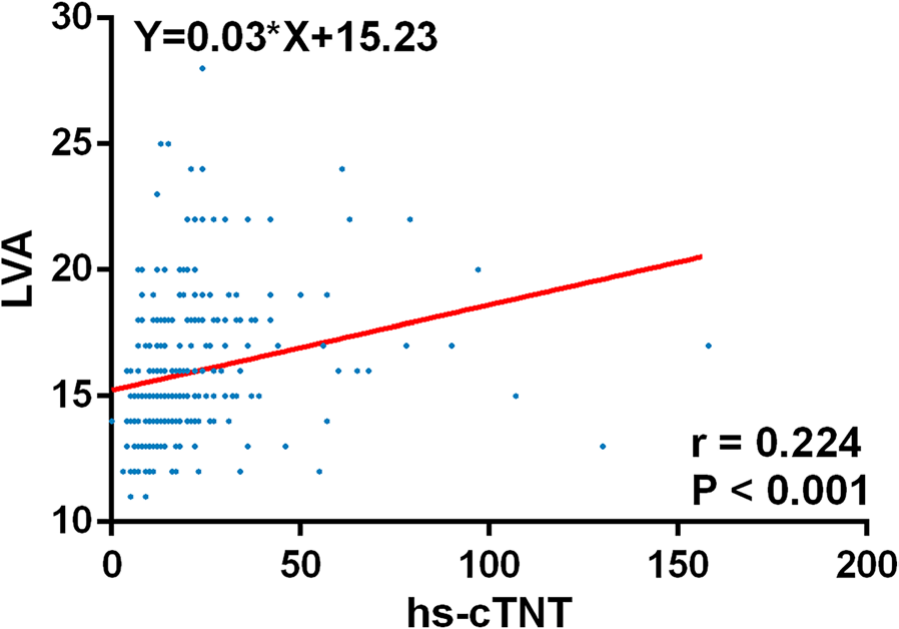
Relationship between LVA and hs-cTnT level

**Fig. 3:**
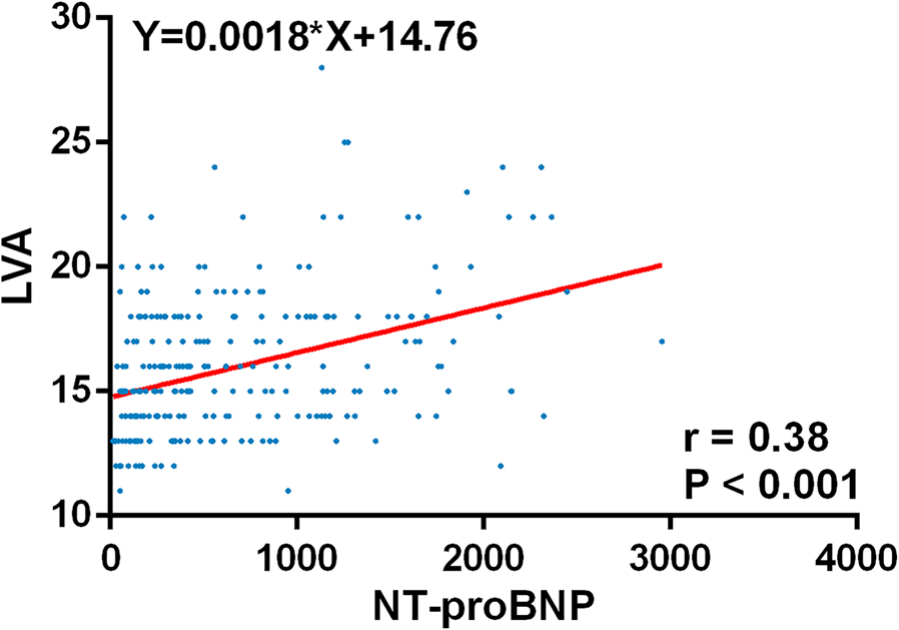
Relationship between LVA and NT-proBNP level

After linear correlation analysis, LVA was both positively linearly correlated with the level of hs-cTNT and NT-proBNP in total patients (hs-cTNT *r* = 0.224, *P* < 0.001; NT-proBNP *r* = 0.38, *P* < 0.001) (Figs. 2 and 3). The Pearson's partial coefficients of correlation between the LVA and level of hs-cTNT, NT-proBNP are shown in Table 2. After adjusted for the factor of age, male, gender, hypertension, smoking, AF, presence of current CAD, creatinine, hemoglobin (Hb), LAD, LVEDD, LVEF and IVS, LVA was also positively correlated with the level of hs-cTNT and NT-proBNP (hs-cTNT adjusted *r* = 0.143, *P* = 0.033; NT-proBNP adjusted *r* = 0.370, *P* < 0.001).

**Table 2:** Linear correlation analysis between hs-cTnT, NT-proBNP levels and LVA

	*r* value	*P* value	Adjusted *r* value	*P* value
Hs-cTNT	0.388	< 0.001	0.143	0.033
NT-proBNP	0.384	< 0.001	0.370	< 0.001

LVA: left ventricular apical thickness; hs-cTNT: high-sensitive cardiac troponin T; NT-proBNP: N-terminal pro-BNP

### Risk factors for hs-cTnT and NT-proBNP levels

Linear regressions were performed to investigate the risk factors for hs-cTNT and NT-proBNP levels (shown in Tables 3 and 4). After multivariable linear regression, the independent risk factors for hs-cTNT levels were creatinine (Beta = 0.199, 95% confidence interval (CI) [0.095, 0.303], *P* < 0.001) and LVA (Beta = 1.182, 95% CI [0.299, 2.064], *P* = 0.009); For the NT-proBNP levels, the independent risk factors were creatinine (Beta = 6.136, 95% CI [3.678, 8.595], *P* < 0.001), LVA (Beta = 61.664, 95% CI [40.736, 82.592], *P* < 0.001), LAD (Beta = 24.021, 95% CI [11.706, 36.335], *P* < 0.001) and LVEDD (Beta = − 22.496, 95% CI [− 39.037, − 5.955], *P* = 0.008). Briefly, LVA was independent risk factor for hs-cTnT and NT-proBNP levels in AHCM patients.

**Table 3:** Risk factors for hs-cTnT level

	Beta (95% CI)	*P* value
Male	− 1.343 (− 8.912, 6.227)	0.727
Age	− 0.055 (− 0.302, 0.193)	0.663
Smoking	1.791 (− 4.186, 7.769)	0.555
Hypertension	− 2.351 (− 7.526, 2.825)	0.372
Diabetes	4.597 (− 1.452, 10.647)	0.136
AF	− 0.945 (− 7.343, 5.454)	0.771
Presence of current CAD	− 3.161 (− 9.865, 3.543)	0.354
HB	− 0.144 (− 0.330, 0.042)	0.128
Creatinine	0.199 (0.095, 0.303)	< 0.001
LVA	1.182 (0.299, 2.064)	0.009
LAD	0.267 (− 0.252, 0.787)	0.312
LVEDD	− 0.514 (− 1.212, 0.184)	0.148
LVEF	0.038 (− 0.518, 0.594)	0.894

AF: atrial fibrillation; CAD: coronary artery disease; HB: hemoglobin; LVA: left ventricular apical thickness; LAD: left atrium diameter; LVEDD: left ventricular end-diastolic diameter; LVEF: left ventricular ejection fraction; hs-cTNT: high-sensitive cardiac troponin T; NT-proBNP: N-terminal pro-BNP

**Table 4:** Risk factors for NT-proBNP level

	Beta (95% CI)	*P* value
Male	− 349.499 (− 528.913, − 170.084)	< 0.001
Age	− 0.600 (− 6.469, 5.269)	0.840
Smoking	− 45.320 (− 187.003, 96.363)	0.529
Hypertension	− 43.932 (− 166.604, 78.741)	0.481
Diabetes	− 57.149 (− 200.535, 86.237)	0.433
AF	237.219 (85.557, 388.882)	0.002
Presence of current CAD	− 67.633 (− 226.540, 91.274)	0.402
HB	− 3.583 (− 7.992, 0.825)	0.111
Creatinine	6.136(3.678, 8.595)	< 0.001
LVA	61.664 (40.736, 82.592)	< 0.001
LAD	24.021 (11.706, 36.335)	< 0.001
LVEDD	− 22.496 (− 39.037, − 5.955)	0.008
LVEF	− 4.067 (− 17.247, 9.113)	0.544

AF: atrial fibrillation; CAD: coronary artery disease; HB: hemoglobin; LVA: left ventricular apical thickness; LAD: left atrium diameter; LVEDD: left ventricular end-diastolic diameter; LVEF: left ventricular ejection fraction; hs-cTNT: high-sensitive cardiac troponin T; NT-proBNP: N-terminal pro-BNP

## Discusion

The retrospective study included AHCM patients and investigated the association between left ventricular apical thickness and cardiac markers, including hs-cTNT and NT-proBNP. The main finding are as follows: 1) Hs-cTNT level abnormal were detected in above half of AHCM patients. 2) our data indicated the strong association between elevated hs-cTNT and NT-proBNP levels with LVA, pointing to the possible influence to structural and functional alterations in AHCM patients.

AHCM is a special form of HCM, with an incidence of approximately 13–25% in Asian populations [10–12]. As a sensitive biomarker of myocardial damage, cardiac troponin was widely used for diagnosis of acute myocardial infarction. In previous studies, cardiac troponin (cTN) level abnormal was found in up to 41–50% in HCM patients [7, 13, 14]. The cTN expression in AHCM has been described in a few studies focusing on the whole population of HCM patients and the results were inconsistent. In a HCM study, which contained 26 AHCM, cardiac troponin I (cTNI) level above 0.04 ng/ml was founded in 34.6% patients [15]. In another research focusing on HCM, hs-cTNT was more likely to be normal in AHCM [16]. However, to the best of our knowledge, there is no specific report on hs-cTNT levels in patients with AHCM. This present study, with a large sample, indicated that up to 51.7% AHCM patients had abnormal hs-cTNT level, which was similar to the percentage in HCM patients.

Approximately 43% to 79% with AHCM patients had chest pain, and AHCM has long been considered a benign disease for a long time [11, 17–19]. However, due to the development of image technology and the awareness of AHCM among clinicians, researchers found it may be associated with adverse cardiovascular events. In a long-term follow-up study, one-third of AHCM patients experienced serious cardiovascular complications, such as myocardial infarction (8.8%) and arrhythmias (17.5%); After follow-up of about 13.6 years, the diameter of LAD increased significantly [8]. In this study, we also found that abnormal hs-cTNT level was associated with higher incidence rate of AF and larger LAD. Another study showed that AHCM was associated with increased mortality, especially in women [18]. In a rarely case, an 18-year-old patient was reported with sudden cardiac arrest and was finally diagnosed with AHCM [20]. This poor prognosis was thought to be associated with subclinical myocyte injury, mainly manifested as elevated cTN. The underlying mechanisms of cTN elevation may be the increased oxygen demand, reduced capillary density and microvascular dysfunction resulting from myocardial hypertrophy [13, 17, 21]. A higher incidence of chest pain has been reported in AHCM patients with myocardial bridging and apical outpouching [22, 23]. To date, no studies have investigated whether the rate of chest pain is elevated in troponin-positive patients. Based on our clinical experience, chest pain may be more common in troponin-positive patients, but further research is required to confirm this.

NT-proBNP is released during increase of ventricular pressure, representing the degree of heart failure. BNP/NT-proBNP was found to be elevated in 86% of HCM patients and it may help stratify the risk of adverse outcomes [7, 24, 25]. NT-proBNP level was also found elevated in AHCM and related to adverse outcomes in a previous study [25]. Shuoyan An and colleagues described that NT-proBNP expression levels in AHCM patients were above normal, although lower than that in HCM patients [26]. Our study showed that NT-proBNP levels were elevated in AHCM patients, especially in those patients with abnormal hs-cTNT level. A study divided AHCM patients into 2 groups according to BNP level and found that LVEDD, left ventricular end-systolic dimension (LVESD), left ventricular fractional shortening and LAD were similar between 2 groups [25]. Interestingly, this present study found NT-proBNP levels were positively correlated with LAD and negatively correlated with LVEDD. The underlying mechanisms and value for prognosis of these findings call for further studies.

Elevated hs-cTNT and NT-proBNP levels portend sustained myocardial infarction and dysfunction, ultimately leading to the changes in cardiac structure and function. High signal-intensity on T2-weighted imaging (HighT2), representing myocardial fibrosis, has been showed to be associated with the elevated hs-cTnT level in cardiovascular magnetic resonance imaging (CMR) study of HCM. And, patients with HighT2 had lager maximal LV wall thickness, LV mass indexed to body surface area (LVMI) and lower LVEF [27]. But it is regrettable that the correlation between HighT2 and IVS, LVA, LVEDD were not described in this study. A study of myocardial contrast echocardiography [28] demonstrated that AHCM had a perfusion defect at the hypertrophied segment. And compared with hypertensive left ventricular hypertrophy (LVH) patients, AHCM patients had larger left atrial volume index (LAVI) and LVEDD. It is well known that apical hypertrophic was the main manifestation of AHCM. However, no previous study described the relationship between cardiac biomarkers and heart structure in AHCM patients to date. For the first time, we demonstrated that serum concentrations of hs-cTNT and NT-proBNP are linearly and positively correlated with LVA, and that LVA is an independent predictor of elevated hs-cTNT in AHCM patients. Therefore, in AHCM patients with elevated hs-cTNT and NT-proBNP levels, it is crucial to maintain effective communication, ensure active follow-up, manage risk factors, and provide optimal medication and best-practice management.

In addition, although our study excluded patients with eGFR levels below 30 mL/(min/1.73 m^2^), we still found that creatinine is an independent predictor of elevated hs-cTnT levels in AHCM patients. Chronic kidney disease (CKD) has consistently been identified as an independent risk factor for both fatal and nonfatal cardiovascular events [29]. Therefore, patients with AHCM combined with renal dysfunction require heightened attention. Type 2 sodium–glucose cotransporter inhibitors (SGLT-2i), a novel class of hypoglycemic agents, have demonstrated benefits for both cardiac and renal health. Juliana et al. confirmed that SGLT-2i alleviates left ventricular hypertrophy by modulating the renin–angiotensin pathway [30]. Whether earlier initiation of SGLT-2 inhibitor therapy is warranted in AHCM patients requires further investigation.

In summary, based on this study, AHCM patients with elevated hs-cTNT or NT-proBNP should be given optimal medication treatment and best management.

## Study limitations

The present study has several limitations. First, this was a single-center and respective study. Second, the outcomes were just the relationship between cardiac biomarkers level and echocardiography parameters in AHCM patients, the long-term follow-up and the influence to prognosis were also important. In addition, previous studies have shown that diastolic function in HCM patients can be impaired [7]. Our data did not include left ventricular diastolic filling pressure, which may affect NT-proBNP levels. However, there is currently no evidence to suggest a relationship between TNT levels and diastolic function, so TNT levels should not be related to diastolic filling pressure. Lastly, our study lacked information on MRI and genetic testing for AHCM patients, which made it impossible to analyze whether elevated troponin levels are associated with gene mutations or small apical aneurysms.

## Conclusions

In this single, retrospective center study, the percentage of abnormal hs-cTNT level was 51.7% in AHCM. LVA was positively correlated with hs-cTNT and NT-proBNP levels in total patients. Depending on the level of hs-cTNT and NT-proBNP, early risk stratification as well as individual therapies could be applied to AHCM.

## Data Availability

The datasets generated during and/or analyzed during the current study are available from the corresponding author on reasonable request.
